# Effects of probiotic supplementation on related side effects after chemoradiotherapy in cancer patients

**DOI:** 10.3389/fonc.2022.1032145

**Published:** 2022-10-28

**Authors:** Yongkai Lu, Xiaoqin Luo, Di Yang, Yi Li, Tuotuo Gong, Binglin Li, Jian Cheng, Ruijuan Chen, Xin Guo, Wei Yuan

**Affiliations:** ^1^ Department of Radiation Oncology, The First Affiliated Hospital of Xi’an Jiaotong University, Xi’an, Shaanxi, China; ^2^ Department of Nutrition and Food Safety, School of Public Health, Xi’an Jiaotong University, Xi’an, China; ^3^ Department of Radiation Oncology, Shaanxi Provincial Tumor Hospital, Affiliated Hospital of Xi’an Jiaotong University Health Science Center, Xi’an, China; ^4^ Department of Obstetrics and Gynecology, Xi'an Central Hospital, The Affiliated Hospital of Xi’an Jiaotong University, Xi’an, Shaanxi, China; ^5^ Department of Neurology, Xijing Hospital, Air Force Medical University, Xi’an, Shaanxi, China

**Keywords:** probiotic, placebo, chemoradiotherapy, cancer, meta-analysis

## Abstract

**Objectives:**

Chemotherapy and radiotherapy generally cause serious adverse side effects in cancer patients, thereby affecting subsequent treatment. Numerous studies have shown that taking probiotics is an option for preventing and treating these side effects. In this investigation, a meta-analysis of the effects of oral probiotics on side effects brought on by radiotherapy, chemotherapy, or chemoradiotherapy treatment will be carried out.

**Methods:**

Two researchers independently and carefully reviewed all pertinent studies that were published before June 30, 2022 and were accessible on PubMed, Embase, Cochrane Library, and the Web of Science. Moreover, the Cochrane Collaboration’s Tool was used to evaluate the risk of bias. Utilizing Review Manager software version 5.4, data were retrieved from eligible studies to evaluate their merits and determine odds ratios (OR) and 95% confidence intervals (CIs) (RevMan 5.4).

**Results:**

2 097 patients from 16 randomized controlled trials were extracted, and standard meta-analysis methods were used to examine the data. Compared with the placebo groups, oral probiotics significantly reduced the side effects caused by radiotherapy and chemotherapy on various types of cancer, such as head and neck cancer, pelvic and abdominal cancer, breast cancer, lung cancer, etc. (OR: 0.31, 95% CI: 0.20 – 0.48; P < 0.005). Further analysis found that the incidence of diarrhea in patients with pelvic and abdominal cancers (OR: 0.32, 95% CI: 0.16 - 0.65; P < 0.005) and the frequency of oral mucositis in patients with head and neck tumors were also significantly lower (OR: 0.28, 95% CI: 0.18 - 0.43; P < 0.005) after the oral administration of probiotics. This suggests that probiotics have a positive influence on the treatment of side effects after chemoradiotherapy. Additionally, a funnel plot revealed that there was no significant publication bias in this study.

**Conclusions:**

Probiotics may help to reduce the occurrence of cancer therapy-related side effects, especially oral mucositis in head and neck tumors and diarrhea in patients with pelvic and abdominal tumors. However, given the small number of clinical trials involved, additional randomized, double-blind, multicentric trials in a larger population are required. This paper may assist researchers in improving trial design in the selection of probiotic strains and selecting appropriate patients who may benefit from probiotic treatments.

## Introduction

The main cause of sickness and mortality in people is cancer. According to GLOBOCAN 2020 predictions, there were roughly 19.3 million new cases and 10 million cancer deaths in 2020, which were much higher than the previous figures from 2018. Both its incidence and mortality are rising quickly globally (1). The two cornerstones of cancer treatment, radiotherapy and chemotherapy, are frequently hindered in their efficacies and applications by their severe side effects, which can include oral mucositis, diarrhea, proctitis, nausea, vomiting, alopecia, cognitive impairment, etc. (2–5). These conditions have a direct impact on patients’ quality of life and may lead to further complications. Developing new cancer treatment strategies has received a lot of attention in recent decades. However, few studies have focused on minimizing the side effects of these cancer treatments.

By 2025, cancer patients must have access to “accurate cancer diagnosis, excellent multimodal therapy, rehabilitation, and supportive and palliative care services,” according to the World Cancer Declaration, which was signed in 2013. To achieve this, safer therapeutic approaches must take into account both therapeutic advantages and accompanying toxicities (6). More researchers have begun to focus on the role of probiotics in preventing or reducing side effects in patients receiving radiotherapy and chemotherapy treatment in recent years. In 2018, a randomized controlled trial of 54 patients with cervical cancer by Linn et al. (7) showed that the incidence of diarrhea in radiotherapy patients in the probiotic group was significantly lower than in the placebo group (53.8% *vs.* 82.1%). Jiang et al. (8) designed a randomized controlled trial to investigate the effect of probiotics in reducing the severity of oral mucositis in patients with nasopharyngeal carcinoma after chemoradiotherapy. According to their research, probiotic combinations greatly improved patients’ immune responses and lessened the severity of oral mucositis *via* altering the gut flora. Recently, Zhang et al. (9) conducted a fascinating study into the application of probiotics. Their team’s randomized double-blind research revealed that probiotic supplements protected chemotherapy-related cognitive impairment in breast cancer patients *via* altering plasma metabolites, such as p-Mentha-1,8-dien-7-ol. Regarding the use of probiotics in colorectal patients after chemotherapy, Babak et al. (10) revealed that supplementation with probiotic preparations improved the quality of life of colorectal patients after chemotherapy, reduced certain inflammatory biomarkers, and relieved some of the side effects of chemotherapy.

Many studies have paved the way for the application of probiotics in the treatment of the side effects of radiotherapy and chemotherapy. However, these studies generally have the problems of small sample size, many research subjects or types of cancer, different outcome indicators, or different bacterial species selection. In other words, even if the subject of the study is one particular type of cancer, the outcome indicators and the bacteria selected may be inconsistent. These problems may lead to the combined analysis of many research results failing to provide clinicians with reliable evidence-based medical proof. As a result, the goal of this study is to collect as many clinical RCT studies as possible for meta-analysis in order to systematically assess the practical application of probiotic supplementation in the treatment of side effects after radiotherapy and chemotherapy in oncology patients and provide a foundation for clinical decision making.

## Methods

### Search methodology

We searched PubMed, Embase, the Cochrane library, and Web of Science for relevant studies published before June 30, 2022 using a combination of medical topic heading (MeSH) phrases and/or free text words including “cancer,” “probiotic,” “placebo,” and “chemoradiotherapy.” There was no restriction on the language of the papers that were published. In addition, we checked the references of the selected studies by hand.Two investigators independently conducted literature searches and screening, and a third investigator was consulted to resolve any differences.

### Inclusion criteria

All included studies adhered to the PICOS guidelines (participants, intervention, comparison, outcomes, and study design). The following were the criteria for inclusion (1): Participants [P]: cancer patients without distant metastases who required radiation or chemotherapy (2); Intervention [I]: probiotics were administered to patients in the experimental group (3); Comparison [C]: control group received placebo (4); Outcomes [O]: the incidence of adverse reactions following radiation or chemotherapy. In order to aggregate the results of as many trials as feasible, we neglected the particular causes of adverse events in this analysis (5); Study design [S]: randomised controlled trials (RCTs) and observational research, such as cohort and case-control studies.

### Criteria for exclusion

Articles conforming to any of the following criteria were excluded (1): Reviews, case reports, correspondence, and abstracts (2); Poor quality or blatantly irrelevant studies were excluded (3); Research without available data that could be combined.

### Extraction of data

Two researchers independently retrieved information from the listed studies (Mr. Yang and Mr. Li): Included in the data were the first author, publication year, age range, cancer kind, probiotic type, results, sample size, and treatment plan. A third investigator arbitrated disagreements concerning data extraction. (Mrs. Guo).

### Quality assessment

Two investigators independently examined and cross-checked the risk of bias in the RCTs (Ms. Guo and Ms. Li). The following characteristics were evaluated using the Cochrane Handbook for Systematic Reviews of Interventions (Version 5.1) to determine the risk of bias: (a) existence of a precise, exact random sequence; (b) use of allocation concealment; (c) anonymisation of researchers and subjects; (d) evaluation of outcomes using a blind procedure; (e) complete data (prevention of probable follow-up loss); (f) study findings are not reported in a biased manner; (g) bias from external sources. These seven biases considered in each study were categorized as “high risk,” “low risk,” or “unclear” if insufficient information was available to determine the possible bias (11).

### Statistical analysis

RevMan software version 5.4 was used to compile the pooled statistics (Cochrane Collaboration, Oxford, UK). The odds ratio (OR) and 95% confidence interval (CI) were selected as the effect indicators to analyze the data on the incidence of adverse events. Using the Cochrane Q test and the *I* ^2^ statistic, which quantified the fraction of total variation attributable to heterogeneity as opposed to chance, heterogeneity between trials was examined (12). If the P-value of the Q test was greater than 0.10 and *I* ^2^ was less than 50 percent, a fixed-effects model was applied to non-significantly heterogeneous data. In the absence of this, a random-effects model was applied to data with substantial variability (13, 14). In addition, a sensitivity analysis was conducted to explore the potential impact of a single study on the entire evaluation. This was accomplished by deleting one study at a time and combining the remaining trials. Furthermore, a funnel plot was utilized to assess the publication’s bias. Publication bias is unlikely if the points in a funnel plot are spread symmetrically on both sides of the dashed center line and are concentrated in the center. Otherwise, there is a strong likelihood of publishing bias.

## Results

### Study selection

**Figure 1:**
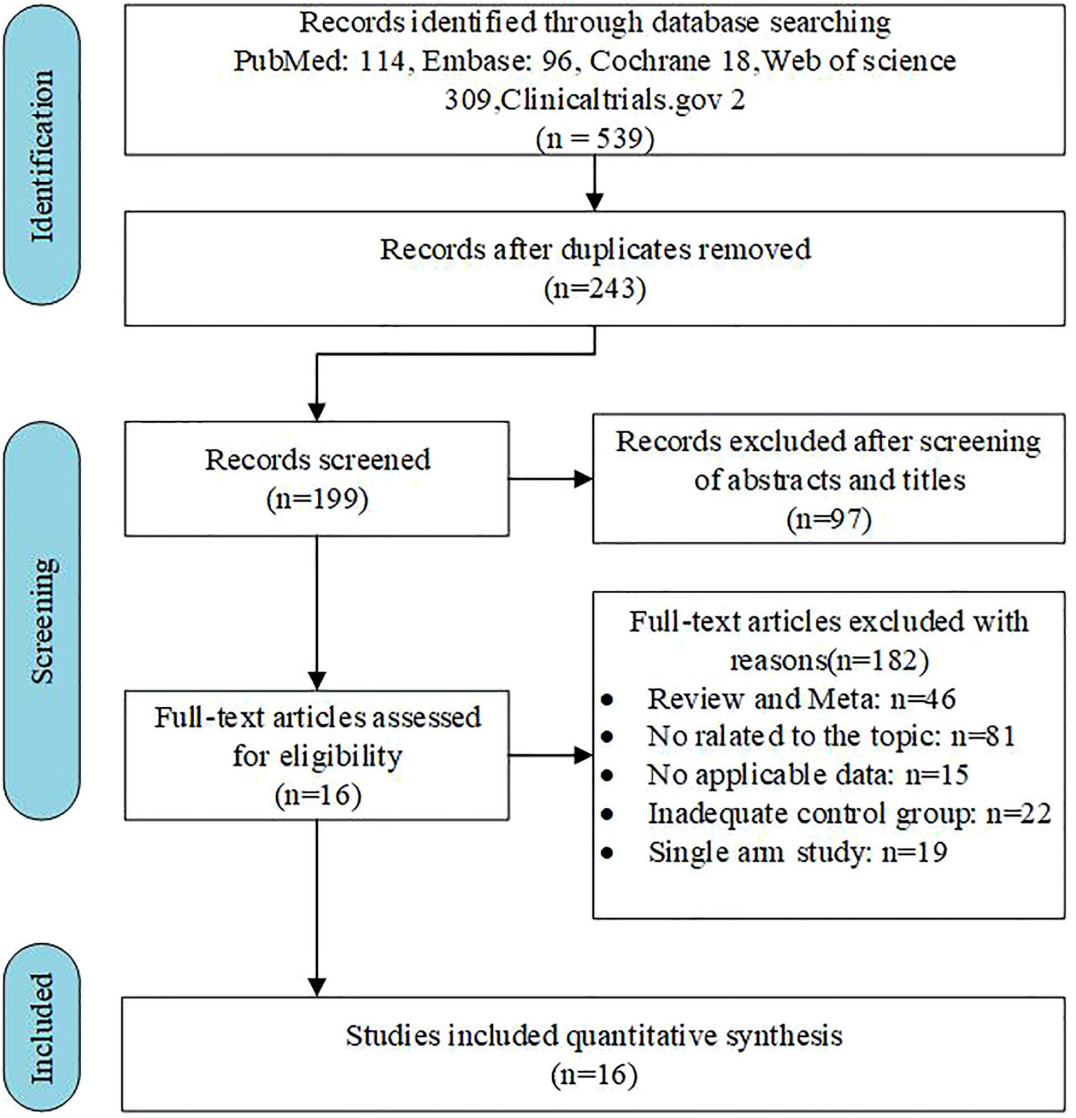
Flow chart of the search process for the meta-analysis.

Initial searches in PubMed, Embase, the Cochrane Library, and Web of Science yielded 396 articles after removing 143 duplicates. After this, 93 articles without the necessary qualifications were weeded out by reading the titles and abstracts. The remaining papers were reviewed in full, and 16 qualified articles were evaluated based on their structure and quality (7–10, 15–26). **Figure 1** depicts the study selection procedure in detail.

### Study characteristics

**Figure 2:**
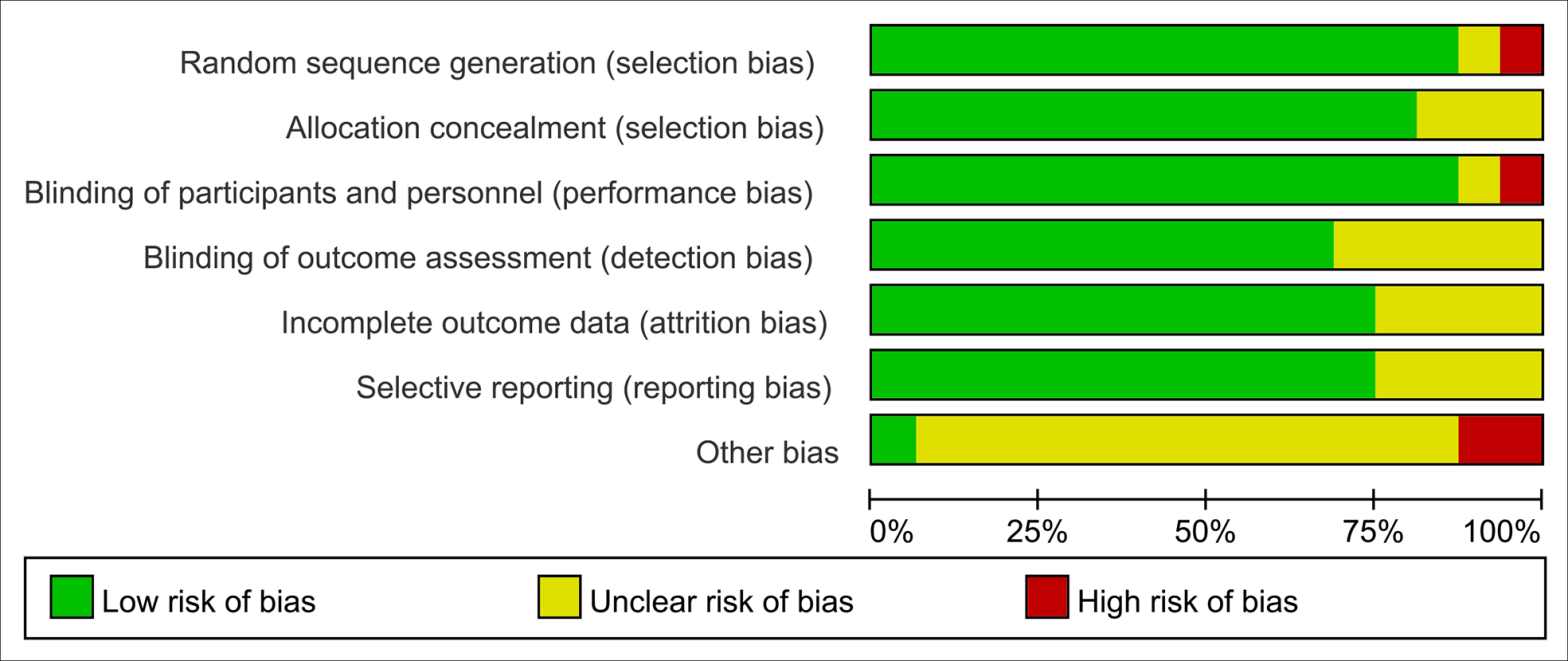
Risk of bias assessment, (risk of bias graph) review authors’ judgements about each risk of bias item presented as percentages across all included studies.

Finally, 16 studies (7–10, 15–26) involving 2,097 cancer patients were included in our meta-analysis. It should be noted that from the statistical data, we classified the outcomes of each study as categorical variables or continuous variables. Only randomized controlled trials with categorical outcomes were included in this study. The papers of Babak et al. (10) and Hilda et al. (26) included outcomes with multiple categorical variables, so in these studies, we only included diarrhea as the study subject. No obvious bias was found in any of the other studies. Eight of the included studies focused on pelvic and abdominal cancers (7, 10, 20, 21, 23–26), five on tumors of the head and neck (8, 15–17, 22), two on breast cancer (9, 18), and one on non-small cell lung cancer (19). In addition, the number and combination of probiotic strains chosen in various clinical trials varied, with four studies (15, 17, 22, 26) using a single strain and the rest studies choosing two or more strains. **Table 1** and **Figure 2** detail the fundamental characteristics and risk of bias evaluation of the included research.

**Table 1:** Characteristics of the studies included in the meta-analysis.

First author (year of publication)	Type of cancer	Types of probiotics	Age range	Total patients(Trail/Control)	Outcomes	Type of treatment	Variable type
Zhang Juan 2022	Breast cancer	Bifidobacterium longum, Lactobacillus acidophilus and Enterococcus faecalis	20-60	159 (80/79)	The incidence of chemotherapy-related cognitive impairment	CT	Categorical
Mirza 2022	Head and neck cancers	Bacillus clausii	30-60	46 (23/23)	The time taken for the appearance, resolution and severity of mucositis, Grade IV, GradeIII	CCRT	Categorical
Chaofei Xia 2021	Nasopharyngeal cancer	*L. plantarum, L. rhamnosus, L. acidophilus*	18-78	70 (36/34)	The incidence of 0, 1, 2, 3, and 4 grades of oral mucositis	CCRT	Categorical
Linn 2018	Cervical cnacer	live *L. acidophilus* LA-5 plus *B. animalis* subsp. *lactis* BB-12	>18	54(26/28)	The incidence of radiation-induced diarrhoea	RT	Categorical
Jiang 2018	Nasopharyngeal cancer	*Bifidobacterium longum, Lactobacillus lactis*, and *Enterococcus faecium*	18-70	93 (58/35)	The incidences of 0, 1, 2, and 3 grades of oral mucositis	CCRT	Categorical
Babak 2017	Colorectal cancer	*L. acidophilus* BCMCR 12130*, L. casei* BCMCR 12313*, Lactobacillus lactis* BCMCR 12451*, Bifidobacterium bifidum* BCMCR 02290*, Bifidobacterium longum* BCMCR 02120 *and Bifidobacterium infantis* BCMCR 02129	>18	140 (70/70)	Quality of life, chemotherapy side effects (diarrhea was included in this study as the research object) and inflammatory markers	CT	Categorical and continuous
Du 2017	Central nervous system tumor	*Bacillus licheniformis*	>3	160 (80/80)	Different grades of nausea, vomiting, abdominal pain, diarrhea and mouth erythema or ulcer	RT	Categorical
Julian 2017	Breast cancer	*L. crispatus* LbV 88 (DSM 22566), *L. rhamnosus* LbV 96 (DSM 22560), *L. jensenii* LbV 116 (DSM 22567), *L. gasseri* LbV 150N (DSM 22583)	53-69	22 (11/11)	Changes in the patient’s vaginal microbiota; Nugent score	CT	Categorical and continuous
Lacouture 2016	Non-small-cell lung cancer	VSL#3 probiotic	≥18	117 (58/59)	The incidence of all-causality; Skindex-16 Scale scores; incidence of all-causality, all grade and grade ≥2 diarrhea; modified-OMDQ scores.	CT	Categorical and continuous
Mego 2015	Colorectal cancer	*Bifidobacterium breve* HA-129 (25%), *Bifidobacterium bifidum* HA-132 HA (20%), *Bifidobacterium longum* HA-135 (14.5%), *Lactobacillus rhamnosus* HA-111 (8%), *Lactobacillus acidophilus* HA-122 (8%), *Lactobacillus casei* HA-108 (8%), *Lactobacillus plantarum* HA-119 (8%), *Streptococcus thermopilus* HA-110 (6%), *Lactobacillus brevis* HA-112 (2%), *Bifidobacterium infantis* HA-116 (0.5%).	≥18	46 (23/23)	Different grades of diarrhea	CT	Categorical
Mimi 2014	Pelvic cancer (gynecologic, rectal, or prostate)	Lactobacillus acidophilus LAC-361 and Bifidobacterium longum BB-536	≥18	167 (81/86)	First appearance of grade ≥2-3-4 diarrhea; incidence in diarrhea (grade ≥ 3)	RT	Categorical and continuous
Sharma 2011	Head and neck cancer	*Lactobacillus brevis* CD2	_	188 (93/95)	The incidence of grade 3 and 4 oral mucositis and the percentage of patients able to complete anticancer treatment.	RT	Categorical
Chitapanarux 2010	Cervical cancer	live *Lactobacillus acidophilus*, Bifidobacterium bifidum	18-65	63 (32/31)	Grade 2 -3 diarrhea.	CCRT	Categorical
Jordi 2007	Gynecologic cancer	*Lactobacillus casei* DN-114 001	≥18	85 (44/41)	Grade 2 or greater diarrhea; quality of life.	RT	Categorical and continuous
Delia 2007	Sigmoid, rectal, or cervical cancer	VSL#3 including four strains of lactobacilli (*L. casei, L. plantarum, L. acidophilus, and L.delbruekii subsp. bulgaricus*), three strains of bifidobacteria (*B. longum, B. breve, and B. infantis*), and one strain of *Streptococcus salivarius subsp. thermophilus*.	_	482 (243/239)	Daily bowel movements and the incidence of radiation-induced diarrhea.	RT	Categorical and continuous
Hilda 2001	Abdomen and pelvis cancer	*Lactobacillus rhamnosus*	19-75	205 (102/103)	Time to and frequency of rescue medication per patient; number of bowel movements; diarrhoea grading (selecteded in this study as the research object); faeces ratings.	RT	Categorical and continuous

CT, chemotherapy; CCRT, radiotherapy plus chemotherapy; RT radiotherapy.

### Overall adverse effects of different cancers

**Figure 3:**
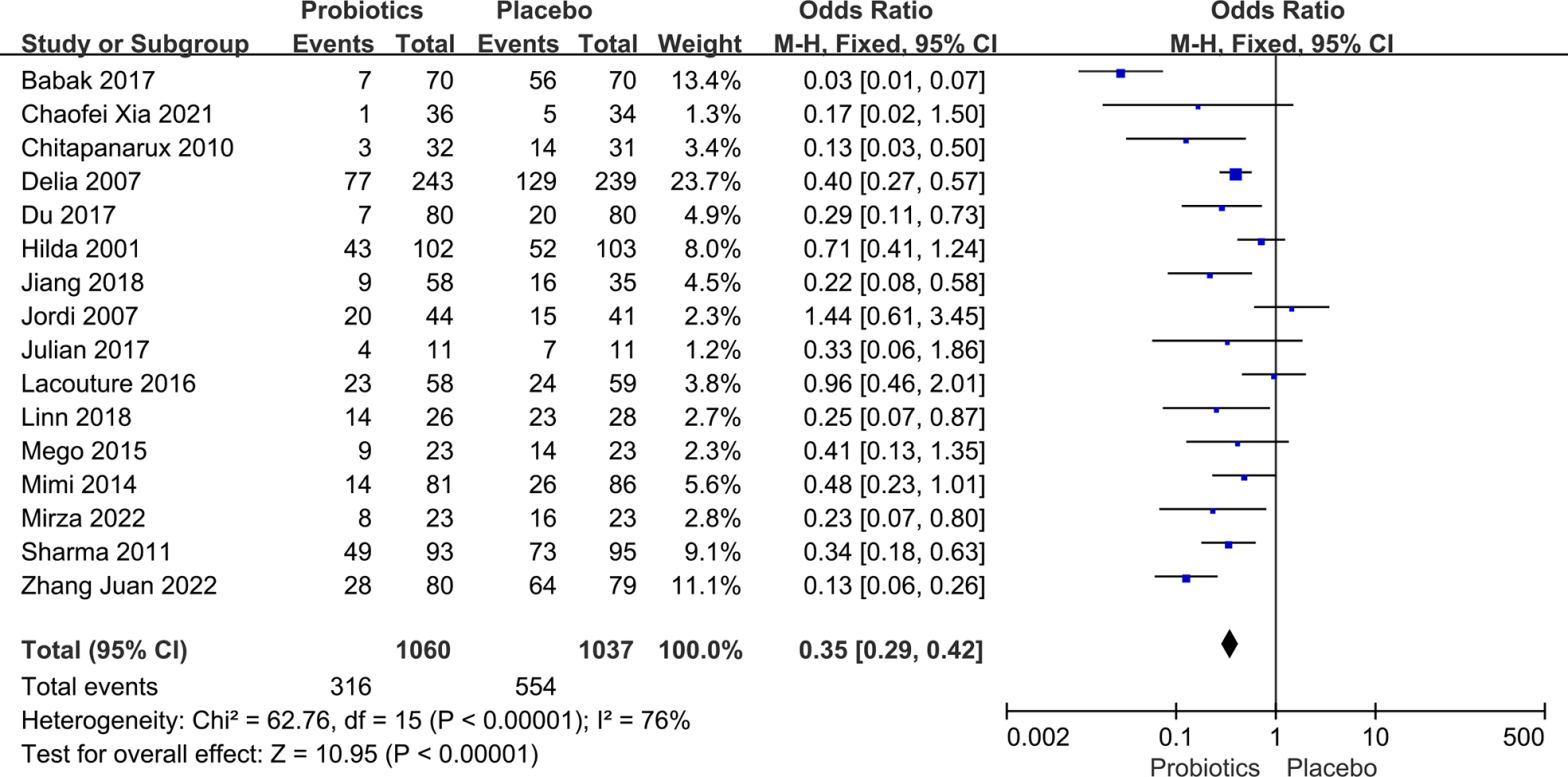
Forest plot of incidence of side effects in probiotic and placebo groups after radiotherapy or chemotherapy for different cancers.

Data on side effects after chemoradiotherapy interventions were extracted from 16 articles including 2,097 patients. Data concerning side effects in this study only included dichotomous variables. In the case of multiple dichotomous variables, the data on the incidence of oral mucositis was preferred for head and neck tumors, and the data on diarrhea for pelvic and abdominal tumors were chosen. Due to the high between-study heterogeneity, the random-effects model was used (*I* ^2^ ≥ 50%, P ≤ 0.10). Pooled results indicated that there was a significant difference between the probiotic group and the placebo group. By merging the results with clinical data from the included trials, we found that probiotic intervention effectively reduced the occurrence of radiation and chemotherapy-related side effects in various malignancies. As **Figure 3** illustrates, the OR, expressed as treatment group *vs.* control group, was 0.31 (95% CI: 0.20 - 0.48; P < 0.005). No side effects due to probiotic administration were reported in any of the literature included in this part of the study.

### Diarrhea for pelvic and abdominal cancers

**Figure 4:**
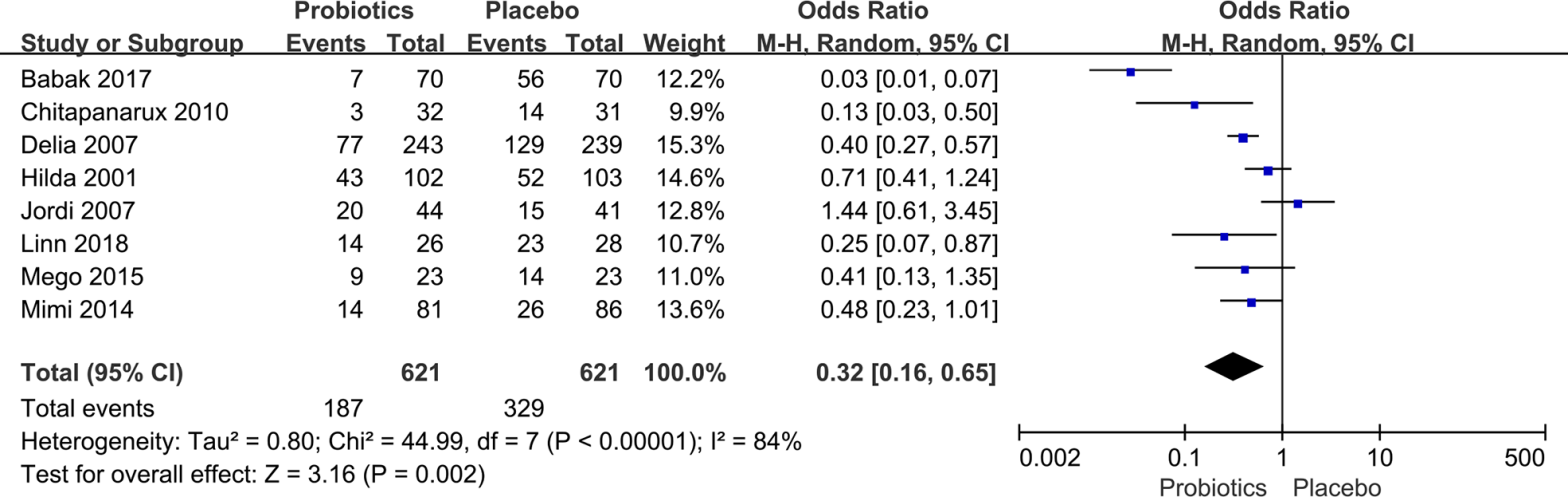
Forest plot of incidence of diarrhea in probiotic and placebo groups after radiation or chemotherapy for pelvic and abdominal cancer.

The incidence of diarrhea after chemoradiotherapy for pelvic and abdominal tumors was extracted from eight studies (7, 10, 20, 21, 23–26) with 1,242 patients. The heterogeneity test revealed statistically significant variations between studies. (*I* ^2^ ≥ 50%, P ≤ 0.10). Thus, a random-effects model was introduced. Compared to the placebo group, pelvic and abdominal cancer patients benefitted greatly from taking probiotics. The data demonstrated that the probability of diarrhea in the probiotic group was significantly lower than in the placebo group (OR: 0.32, 95% CI: 0.16 - 0.65; P < 0.005; **Figure 4**). In any of the literature that was looked at for this part of the study, there were no reports of side effects from taking probiotics.

### Oral mucositis in head and neck cancer

**Figure 5:**
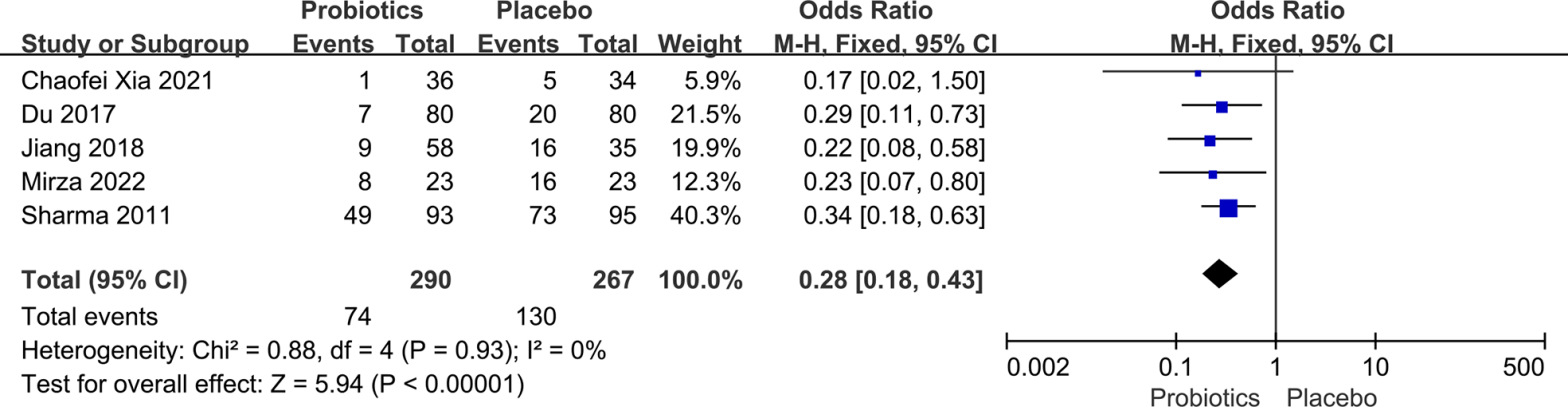
Forest plot of the incidence of oral mucositis in the probiotic and placebo groups after radiation or chemotherapy for head and neck cancer.

Five studies (8, 15–17, 22) involving 557 head and neck cancer patients were included in this analysis. During the analysis, we discovered no substantial heterogeneity between studies (*I* ^2^ = 0%; P = 0.93), so a fixed-effects model was used. Compared to placebo, the use of probiotics decreased the incidence of oral mucositis in patients with head and neck cancer who had chemoradiotherapy (OR: 0.28, 95% CI: 0.18 - 0.43; P < 0.005; **Figure 5**). There were no reports of side effects from probiotic administration in any of the literature examined in this section of the study.

### Senstivity analysis

Our primary analysis results, as well as those of our sensitivity studies, were not significantly different from one another. The findings of sensitivity analyses for the three indicators of overall bad effects in various malignancies, diarrhea in pelvic and abdominal cancers, and oral mucositis in head and neck cancers, are presented in Appendix **Figures S1, S2**, and **S3**, respectively.

### Publication bias

**Figure 6:**
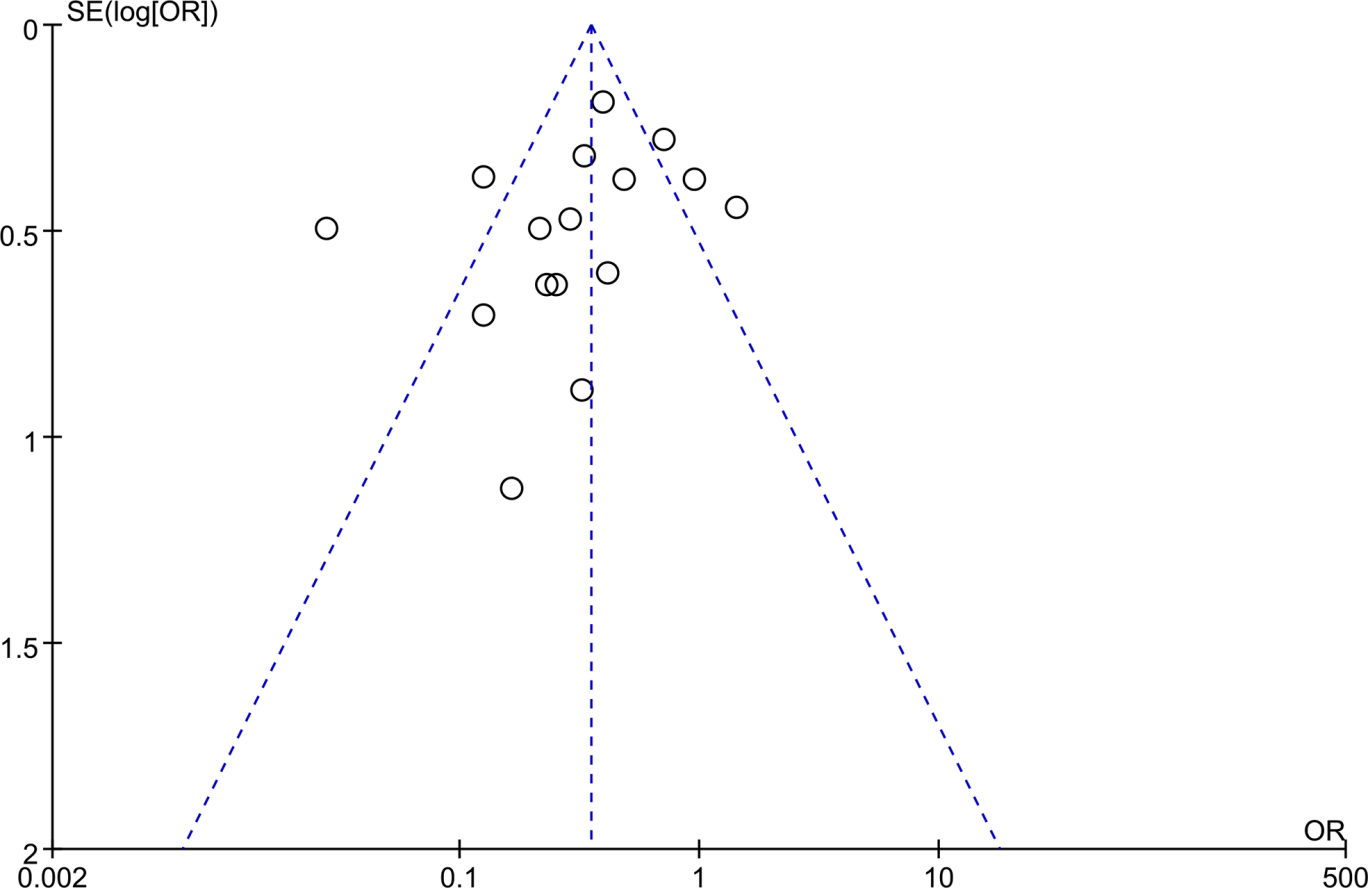
Funnel plots for potential publication bias.

Utilizing a funnel plot, publication bias in the literature was evaluated. If there were at least 10 studies included in the meta-analysis, funnel plot asymmetry tests were conducted (27). The funnel plot of various indicators (**Figure 6**) demonstrates that the point estimates are symmetrically distributed on both sides and centered in the middle. Because of this, there was no proof of publishing bias.

## Discussion

### Main findings

In this study, we identified 16 RCTs that assessed the use of probiotics in the prevention of chemoradiotherapy-induced adverse events. Of these, eight papers reported on diarrhea data in patients with pelvic and abdominal cancers, five studies provided oral mucositis information on patients with head and neck tumors, two RCTs focused on adverse effects in breast cancer patients, and one paper described the side effects after chemoradiotherapy in non-small cell lung cancer patients. In terms of treatment tactics (strains, dosages, and probiotic therapy duration), patient ages, comorbidities, tumor kinds, and measured outcomes, these investigations were varied. This may explain the between-study heterogeneity of the results. For studies where meta-analyses were possible, sensitivity analyses showed no qualitative change in conclusions when changes between studies were assessed. Due to the limited number of heterogeneous studies, subgroup analysis were unable to be conducted. Ultimately, for all cancers included in this study, probiotics showed modest beneficial effects in reducing various side effects of chemoradiotherapy. A more comprehensive investigation revealed that probiotics significantly decreased oral mucositis in individuals with head and neck cancers and diarrhea in patients with pelvic-abdominal tumors following chemoradiotherapy. Consequently, probiotic supplementation may be considered a viable adjuvant therapy for patients who have had radiotherapy and chemotherapy.

### Comparison with previous meta-analyses

In the past five years, three high-quality meta-analyses (28–30) have studied the effectiveness of probiotics in avoiding radiation and chemotherapy-related adverse effects in cancer patients. Feng et al. (28) conducted a pooled study on the incidence of oral mucositis and diarrhea after chemotherapy. Also, Wang et al. (30) studied the incidence of diarrhea after chemoradiotherapy for pelvic and abdominal tumors. Shu et al. (29) also focused on oral mucositis. In contrast, our meta-analysis included more randomized controlled studies with a total of 2,097 cancer patients from 16 publications. Additionally, our research was more diverse and we did not differentiate between the various forms of cancer, types of side effects, and treatments. In terms of treatment methods, cancer treatment is increasingly moving towards comprehensive treatment plans. This means it is necessary to have a deeper understanding of the side effects of radiotherapy and chemotherapy treatment, rather than to study them separately. Finally, the conclusions of this paper are more specific than in existing studies. Firstly, probiotics have a positive effect on most side effects reported after chemoradiotherapy. Moreover, they effectively prevent oral mucositis and diarrhea.

### Efficacy

In a pooled analysis of all included studies (7–10, 15–26), each trial studied a variety of outcome indicators. Generally speaking, the indicators can be divided into two categories according to the variable type. One is categorical variables, which mainly include the incidence of certain adverse events, while the other is continuous variables, which comprise occurrence time, cut-off time, and adverse event severity score. Our meta-analysis revealed that for adverse event rates (binary variables), the probiotic group exhibited a significant reduction in side effects (OR: 0.31, 95% CI: 0.20 - 0.48; P < 0.005). Additionally, this summary included heterogeneous factors such as multiple cancer types (e.g., head and neck tumors, pelvic and abdominal tumors, breast cancer, lung cancer), multiple adverse reaction indicators (e.g., diarrhea, mucositis, cognitive impairment, vaginal microbiota changes), and multiple treatment methods (radiotherapy or chemotherapy), and results suggest that the probiotic group is generally superior to the placebo group. The “generally useful” effect of probiotics is explained in a review on the mechanism of probiotics published by Oelschlaeger (31) in 2010. Here, probiotics may modify host defenses, and this method of action is expected to promote the prevention and treatment of inflammation in the digestive tract or its components. In addition, probiotics have direct effects on commensal and/or pathogenic microbes.This idea is crucial for avoiding and treating infections and restoring the microbial balance in the gut in many instances (31).

Studies have also shown that radiation causes disruption of the gut microbiome, alters bacterial flora, disrupts host homeostasis, damages gut microvilli, decreases enzymatic activity, and reduces overall gut transit time, ultimately leading to diarrhea (21, 32–37). Moreover, a number of *in vitro* and *in vivo* research demonstrated that chemotherapeutic medicines trigger death in crypt cells, leading to decreased intestinal absorption, altered gut microbiota, impaired gut homeostasis, and ultimately diarrhea (38–40).. In our research, the incidence of diarrhea, defined as the frequency of Grade 3 or 2 diarrhea, was used as the primary outcome. Regarding the incidence of diarrhea in patients with abdominal and pelvic malignancies, this meta-analysis demonstrated a significant reduction in the probiotic group (OR: 0.32, 95% CI: 0.16 - 0.65; P < 0.005). Additionally, we found that among the eight studies (7, 10, 20, 21, 23–26) that included diarrhea data, the results of Urbancsek et al. (26) and Giralt et al. (24) showed larger OR values, at 0.71 and 1.44, respectively. This indicated that the advantage of the probiotic group was not obvious or even nonexistent, which may be related to the fact that they only selected a single species of Lactobacillus rhamnosus or Lactobacillus casei DN-114 001 in clinical trials. In the other studies (7, 10, 20, 21, 23, 25), using multiple probiotic combinations, the probiotic groups showed a significant reduction in diarrhea. These findings are consistent with the study by Feng et al. (28).

In clinical practice, the occurrence of oral mucositis after chemoradiotherapy in patients with head and neck tumors is essentially unavoidable. We included a meta-analysis of five studies involving 557 participants to evaluate the effectiveness of probiotics in the prevention and treatment of oral mucositis caused by cancer treatments, including chemotherapy, radiotherapy, and chemoradiotherapy. Results of the pooled analysis showed that probiotic intervention effectively reduced the incidence of oral mucositis (OR: 0.28, 95% CI: 0.18 - 0.43; P < 0.005). Additionally, there was no significant heterogeneity (*I* ^2^ = 0, P = 0.93) among the five clinical trials. Interestingly, three researchers (15, 17, 22) used a single strain of probiotic, while two (8, 16) used a combination of multiple strains, but their results all showed that the probiotic group exhibited obvious advantages. These findings contradict the conclusion that a single probiotic strain is ineffective against diarrhea in patients with pelvic and abdominal tumors.

### Safety

Systemic infections, detrimental metabolic activity, excessive immunological activation, gene transfer, obesity, skin problems, and gastrointestinal side effects have been linked to probiotic use (41).. Due to the heterogeneity of different treatment regimens and malignancies, probiotic-related side effects could not be distinguished. None of the studies included in this paper reported adverse effects caused by probiotics, but Giralt et al. (24) claimed that taking probiotics did not improve post-radiation diarrhea in gynecological cancer patients. Of the 41 patients in the placebo group, 24 suffered Grade 2 or higher diarrhea, while 30 of the 44 patients in the probiotic group had Grade 2 or higher diarrhea. In conclusion, based on the available evidence, we are still unable to estimate whether it is safe for cancer patients to receive probiotics for various side effects due to the numerous variables involved, such as probiotic strain type, dosage, duration of use, and suitability of patients with the right physical condition.

### Strengths and weaknesses

Despite the fact that our results demonstrate that probiotics can lessen various adverse effects of cancer treatment, including oral mucositis and diarrhea, it is important to note some potential limits. First, there are few studies available. Besides, the participants in the study were diagnosed with different types of cancer, which may affect the reliability of the final results. Also, there are no clinical standards for the active ingredients and dosage of probiotics, which leads to different experimental protocols in each study. Finally, most of the studies focused on head and neck, pelvic, and abdominal tumors, with only two studies concerning breast cancer and one study involving lung cancer, making the findings less general. However, since this is a more comprehensive and systematic meta-analysis of the topic, the results are meaningful and clinically valuable. Microorganisms have recently been shown to play an important role in many diseases (42, 43). As with the aforementioned constraints, the choice and mix of probiotics and target demographics varies from study to study, making it challenging to suggest and approve them in clinical usage guidelines. This study may help researchers to select appropriate probiotics and extrapolate their potentially beneficial effects on patients, especially head and neck, pelvic, and abdominal tumors.

## Conclusions

This meta-analysis reveals that probiotics may lower the occurrence of side effects caused by cancer therapy, particularly oral mucositis in patients with head and neck malignancies and diarrhea in individuals with pelvic and abdominal tumors. Due to the modest number of clinical trials included in this study, however, additional randomized, double-blind, multicentric trials in a broader population are necessary. This work assists researchers in improving the design of clinical trials involving the selection of probiotic strains and the selection of patients who may benefit from probiotic therapy.

## Data availability statement

The original contributions presented in the study are included in the article/**Supplementary Material**. Further inquiries can be directed to the corresponding authors.

## Author contributions

YKL, DY, and XL: Conceptualization. YL, TG, BL: Data curation and original draft writing. RC, XG, WY: Statistical analysis. YL, JC: Manuscript review and editing. All authors contributed to the article and approved the submitted version.

## Funding

Xi'an Innovation Capability Strong Foundation Project No. 21XYJ0021; Xi'an Central Hospital Scientific Research Project No. 2022QN06.

## Conflict of interest

The authors declare that the research was conducted in the absence of any commercial or financial relationships that could be construed as a potential conflict of interest.

## Publisher’s note

All claims expressed in this article are solely those of the authors and do not necessarily represent those of their affiliated organizations, or those of the publisher, the editors and the reviewers. Any product that may be evaluated in this article, or claim that may be made by its manufacturer, is not guaranteed or endorsed by the publisher.
