# Supplementary material for: Effects of probiotic supplementation on related side effects after chemoradiotherapy in cancer patients
Source: Front Oncol. 2022 Oct 28;12:1032145. doi: 10.3389/fonc.2022.1032145 (PMC9650500; doi:10.3389/fonc.2022.1032145)
Supplement: Supplementary file 1 [file Table_1.docx]

ID Search

#1 MeSH descriptor: [Probiotics] explode all trees

#2 (Probiotic):ti,ab,kw (Word variations have been searched)

#3 MeSH descriptor: [Placebos] explode all trees

#4 (Sham Treatment):ti,ab,kw (Word variations have been searched)

#5 MeSH descriptor: [Neoplasms] explode all trees

#6 (cancer):ti,ab,kw OR (Tumor):ti,ab,kw OR (Neoplasm):ti,ab,kw OR (Tumors):ti,ab,kw OR (Neoplasia):ti,ab,kw (Word variations have been searched)

#7 (Neoplasias):ti,ab,kw OR (Cancers):ti,ab,kw OR (Malignant Neoplasm):ti,ab,kw OR (Malignancy):ti,ab,kw OR (Malignancies):ti,ab,kw (Word variations have been searched)

#8 (Malignant Neoplasms):ti,ab,kw OR (Neoplasm, Malignant):ti,ab,kw OR (Neoplasms, Malignant):ti,ab,kw OR (Benign Neoplasms):ti,ab,kw OR (Benign Neoplasm):ti,ab,kw (Word variations have been searched)

#9 (Neoplasms, Benign):ti,ab,kw OR (Neoplasm, Benign):ti,ab,kw (Word variations have been searched)

#10 #1 OR #2

#11 #3 OR #4

#12 #5 OR #6 OR #7 OR #8 OR #9

#13 #10 and #11 and #12

#14 MeSH descriptor: [Radiotherapy] explode all trees

#15 (Radiotherapies):ti,ab,kw OR (Radiation Therapy):ti,ab,kw OR (Radiation Therapies):ti,ab,kw OR (Therapies, Radiation):ti,ab,kw OR (Therapy, Radiation):ti,ab,kw (Word variations have been searched)

#16 (Radiation Treatment):ti,ab,kw OR (Radiation Treatments):ti,ab,kw OR (Treatment, Radiation):ti,ab,kw OR (Radiotherapy, Targeted):ti,ab,kw OR (Radiotherapies, Targeted):ti,ab,kw (Word variations have been searched)

#17 (Targeted Radiotherapies):ti,ab,kw OR (Targeted Radiotherapy):ti,ab,kw OR (Targeted Radiation Therapy):ti,ab,kw OR (Radiation Therapies, Targeted):ti,ab,kw OR (Targeted Radiation Therapies):ti,ab,kw (Word variations have been searched)

#18 (Therapies, Targeted Radiation):ti,ab,kw OR (Therapy, Targeted Radiation):ti,ab,kw OR (Radiation Therapy, Targeted):ti,ab,kw (Word variations have been searched)

#19 MeSH descriptor: [Chemoradiotherapy] explode all trees

#20 (Chemoradiotherapies):ti,ab,kw OR (Radiochemotherapy):ti,ab,kw OR (Radiochemotherapies):ti,ab,kw OR (Concurrent Chemoradiotherapy):ti,ab,kw OR (Chemoradiotherapies, Concurrent):ti,ab,kw (Word variations have been searched)

#21 (Chemoradiotherapy, Concurrent):ti,ab,kw OR (Concurrent Chemoradiotherapies):ti,ab,kw OR (Synchronous Chemoradiotherapy):ti,ab,kw OR (Chemoradiotherapies, Synchronous):ti,ab,kw OR (Chemoradiotherapy, Synchronous):ti,ab,kw (Word variations have been searched)

#22 (Synchronous Chemoradiotherapies):ti,ab,kw OR (Concurrent Radiochemotherapy):ti,ab,kw OR (Concurrent Radiochemotherapies):ti,ab,kw OR (Radiochemotherapies, Concurrent):ti,ab,kw OR (Radiochemotherapy, Concurrent):ti,ab,kw (Word variations have been searched)

#23 (Concomitant Chemoradiotherapy):ti,ab,kw OR (Chemoradiotherapies, Concomitant):ti,ab,kw OR (Chemoradiotherapy, Concomitant):ti,ab,kw OR (Concomitant Chemoradiotherapies):ti,ab,kw OR (Concomitant Radiochemotherapy):ti,ab,kw (Word variations have been searched)

#24 (Concomitant Radiochemotherapies):ti,ab,kw OR (Radiochemotherapies, Concomitant):ti,ab,kw OR (Radiochemotherapy, Concomitant):ti,ab,kw (Word variations have been searched)

#25 MeSH descriptor: [Drug Therapy] explode all trees

#26 (Therapy, Drug):ti,ab,kw OR (Drug Therapies):ti,ab,kw OR (Therapies, Drug):ti,ab,kw OR (Chemotherapy):ti,ab,kw OR (Chemotherapies):ti,ab,kw (Word variations have been searched)

#27 (Pharmacotherapy):ti,ab,kw OR (Pharmacotherapies):ti,ab,kw (Word variations have been searched)

#28 #14 OR #15 OR #16 OR #17 OR #18 OR #19 OR #20 OR #21 OR #22 OR #23 OR #24 OR #25 OR #26 OR #27

#29 #13 AND #28
