# Supplementary figures and images for: Effects of probiotic supplementation on related side effects after chemoradiotherapy in cancer patients
Source: Front Oncol. 2022 Oct 28;12:1032145. doi: 10.3389/fonc.2022.1032145 (PMC9650500; doi:10.3389/fonc.2022.1032145)

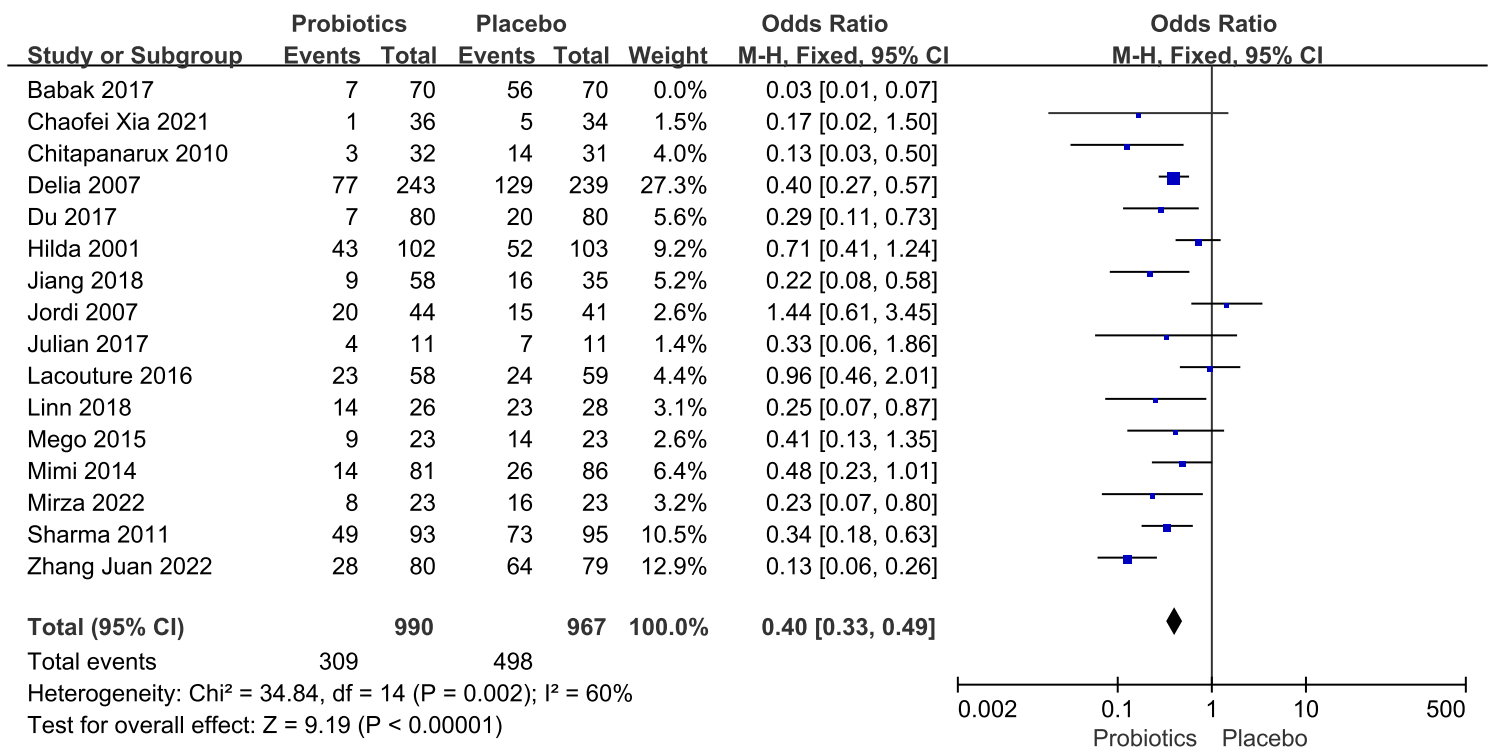

Supplement: Supplementary file 2 [file Presentation_1.zip › APPENDIX figure/S1.pdf]

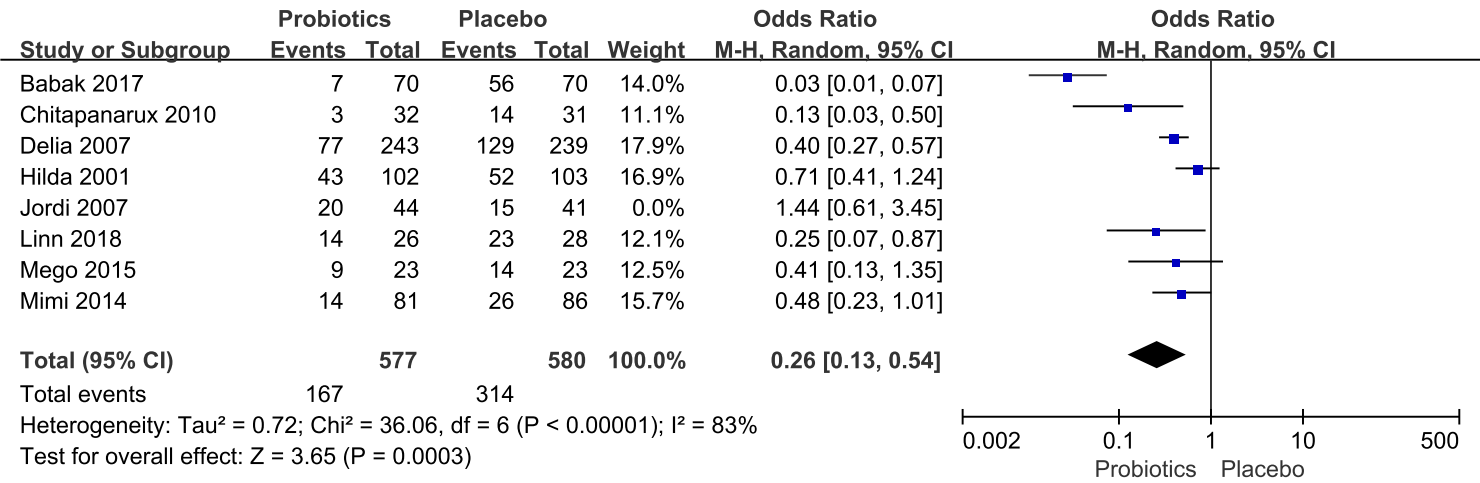

Supplement: Supplementary file 2 [file Presentation_1.zip › APPENDIX figure/S2.pdf]

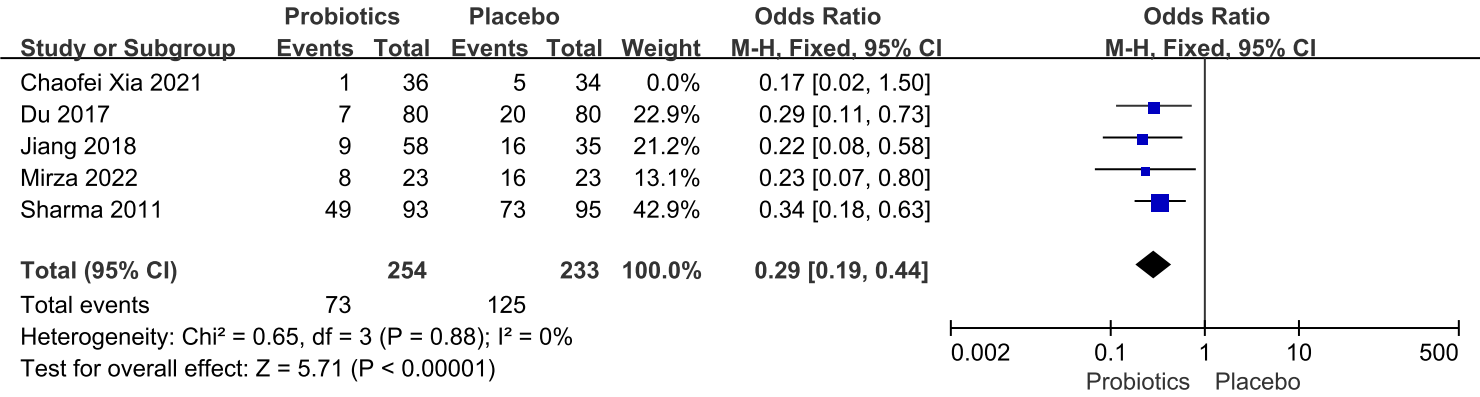

Supplement: Supplementary file 2 [file Presentation_1.zip › APPENDIX figure/S3.pdf]
